# Beta-blockers in post-acute myocardial infarction patients: Drug prescription patterns from 2018 to Italy’s first wave of the COVID-19 pandemic

**DOI:** 10.3389/fphar.2022.1040710

**Published:** 2022-12-07

**Authors:** Elena Olmastroni, Federica Galimberti, Alberico L. Catapano, Elena Tragni, Manuela Casula

**Affiliations:** ^1^ Epidemiology and Preventive Pharmacology Service (SEFAP), Department of Pharmacological and Biomolecular Sciences, University of Milan, Milan, Italy; ^2^ IRCCS MultiMedica, Sesto S. Giovanni, MI, Italy

**Keywords:** COVID-19 pandemic, beta-blockers, acute myocardial infarction, cardiovascular diseases, appropriate drug prescription

## Abstract

**Background:** Major guidelines recommend the initiation of a beta-blocker therapy after an acute myocardial infarction (AMI). We aimed to map the treatment pathway of beta-blockers for AMI survivors during the first wave of COVID-19 pandemic in Italy and to investigate predictors for treatment non-initiation.

**Methods:** Healthcare utilization databases of Lombardy Region were investigated. Subjects aged ≥18 years who were hospitalised with AMI in the period February-March-April of 2018, 2019, and 2020 were included, and followed for 30 days from the discharge date, to investigate whether they presented a first prescription of beta-blockers. A multivariate logistic model was performed to evaluate the effect of several covariates on the probability of not receiving a post-AMI beta-blocker therapy.

**Results:** The cohorts comprised 2259, 2383, and 1932 individuals who were hospitalised with AMI in the 3-month period in 2018, 2019, and 2020, respectively. Overall in 2020, about 58–60% of individuals with AMI received a prescription of beta-blockers within 1 month after the discharge. A continuous decreasing trend over time was observed. Men were 30% more likely to start the treatment than women, increasing age was associated with significant increasing probability of not receiving a post-infarction beta-blocker therapy, while having received an antihypertensive or lipid-lowering treatment, or having been hospitalized for heart failure prior to the AMI hospitalization reduced the likelihood of not being treated with beta-blockers.

**Conclusion:** The initiation of beta-blocker treatment after AMI remains an under-prescribed practice, that does not seem to have been further affected by the first wave of the COVID-19 pandemic.

## Introduction

Patients with acute myocardial infarction (AMI) are a high-risk group with increased mortality; thus, the current practice guidelines emphasize the importance of intensive risk factor modification in patients with previous AMI (Amsterdam et al., 2014).

Beta-blockers have improved survival and are one of the cornerstones in the treatment of ischemic heart disease; they exert an antianginal effect by reducing the myocardial workload and oxygen demand (Yusuf et al., 1985). Besides, they also have antiarrhythmic and anti-remodeling effects (Olsson and Rehnqvist, 1986). Treatment with beta-adrenergic blocker therapy after an AMI reduces infarct size and decreases the risk of recurrent AMI and death when started early (Beta-Blocker Heart Attack Study Group, 1981; Yusuf et al., 1988; Raposeiras-Roubin et al., 2015).

Although there is some debate in the recent literature about their long-term use (Zeitouni et al., 2019), the start of beta-blocker therapy in the post-AMI setting have been recommended by guidelines for years (Amsterdam et al., 2014; Roffi et al., 2016; Ibanez et al., 2017). The most recent indications by the major scientific societies in secondary cardiovascular prevention also confirm that routine administration of beta-blockers in all post-AMI patients should be considered, while are recommended in patients with reduced left ventricular ejection fraction (LVEF ≤40%) (Collet et al., 2021; Visseren et al., 2021), unless contraindicated, as in case of active bronchospasm or hypotension (Levine et al., 2016).

Although preventive medication reduces mortality, prescribing and adherence are known to be frequently insufficient. Findings from various studies indicate considerable underuse of beta-blockers following myocardial infarction, with only 20 to 50 percent of eligible patients receiving these agents. Underutilization of beta-blockers may be attributed, in part, to the fear of adverse effects, especially in the elderly and in patients with concomitant disorders (Fonarow, 2005).

As already described for other prescribing performance indicators (Bezzini et al., 2021; Ismail et al., 2022; Villalobos Violan et al., 2022), the initial spread of coronavirus disease 2019 (COVID-19) pandemic, in which government has implemented various initiatives to prevent or delay the spread of COVID-19, may have worsened this situation. Indeed, the emergency situation may have adversely affected the physician’s attention to good prescribing practices. The spread of the infection, together with the initial lack of knowledge of its aetiology, may have led doctors to under-prescribe new drugs assuming that an increased drug load could put patients at greater risk. This was compounded by patients’ fear of visiting doctors’ offices and pharmacies.

The purpose of this analysis was to map the treatment pathway of beta-blockers for AMI survivors before and during the first wave of COVID-19 pandemic in Italy, especially during the period between February and April 2020, with a full national lockdown, to evaluate age and sex differences in pharmaceutical treatment initiation after discharge, and to investigate potential predictors for treatment non-initiation.

## Methods

### Data source

Data used in this study were retrieved from the healthcare utilization databases of Lombardy Region (data availability 2017–2020), in particular: 1) the archive of Lombardy’s residents with a coverage from the Italian NHS, containing demographic variables (sex, date of birth, date of death); 2) the drugs’ prescription archive, including information on the drugs reimbursed by NHS delivered from any pharmacy in the Region, as the corresponding Anatomical Therapeutic Chemical (ATC) code and the prescription date, and 3) the hospital discharge archive recording, among others, the admission date and primary and secondary diagnoses of all hospitalizations at public or private hospitals of the Region.

The data belonging to each subject stored in different archives are linkable using a subject’s unique encrypted identification key allowing the reconstruction of the medical history of each individual belonging to the target population. The identification key was appropriately encrypted to prevent the identification of the subjects, as for the European Regulation No. 2016/679 and the national Legislative Decree 101/2018.

### Study population

All beneficiaries of the NHS, resident in Lombardy, of both sexes, with age ≥18 years, who were hospitalised with AMI in the period February-March-April 2020 (index date) were included in the cohort. To identify individuals hospitalised with AMI from the hospital discharge database, we searched for ICD-9 diagnosis code “410, 3606, 3610, 3611, 3612, 3613, 3614, 0066” in the main diagnosis, concomitant diagnoses, diagnostic or therapeutic procedures. Subjects who had already been treated with beta-blockers (ATC “C07, C09BX02, C09BX04, C09BX05, C09DX05”) in the 6 months prior to the hospitalisation or presented particular conditions such as asthma or chronic obstructive pulmonary disease (ATC “R03AC, R03AK, R03BA, R03BB, R03DC” or ICD-9 diagnosis code “493, 491, 492”), or peripheral vascular disease (ICD-9 diagnosis code “443, 459”) for which beta-blockers are contraindicated (Chen et al., 2001), were excluded.

Each selected patient was followed for 30 days from the discharge date, to investigate whether or not patients presented a first prescription of beta-blockers; in a sensitivity analysis, the follow-up period was extended to 6 months. Subjects who had a follow-up shorter than 6 months were excluded.

### Patient characteristics

The information on several potential confounding factors was collected at the index date or in a period of time before the index date. In particular, subjects’ age and sex were assessed at the index date, the use of antiplatelet (ATC “B01AA, B01AC, B01AE, B01AF, B01AX”), antihypertensive (ATC “C02, C03, C08, C09”, excluding beta-blockers), lipid-lowering (ATC “C10AA, C10BA”), and antidiabetic (ATC “A10B”) drugs was evaluated in the 6 months before the index date, while the occurrence of hospitalizations for heart failure (HF, ICD-9 diagnosis code “39891, 40211, 40291, 40411, 40413, 40491, 40493, 428”) were evaluated in the 12 months before the index date.

### Statistical analysis

Continuous variables are expressed as mean and standard deviation (SD) while categorical data as absolute frequencies and percentages.

We initially assessed the percentage of subjects who started treatment with beta-blockers within 30 days after an AMI by each month of the period February-March-April 2020. We then compared rates with those of similar cohorts of subjects enrolled in the same months in the years 2019 and 2018. Stratified analyses were performed by sex and age classes (18–49, 50–64, 65–84, ≥85 years) to assess if pattern use varied among strata.

A multivariate logistic regression analysis was conducted to evaluate the effect of several variables (sex, age, antiplatelet, antihypertensive, lipid-lowering, and antidiabetic treatments, and HF hospitalization) on the probability of not receiving a post-AMI beta-blocker therapy, among individuals hospitalised with AMI in the period February-March-April 2018–2020. Model estimates are presented as odds ratios (OR) and the corresponding 95% confidence interval (95%CI).

Data analysis was performed using SAS (Statistical Analysis System) software version 9.4 (SAS. Institute, Inc. Cary, North Carolina), and two-tailed *p* < 0.05 was considered for statistical significance in all analyses.

## Results

The cohorts comprised 2259, 2383, and 1932 individuals who were hospitalised with AMI in the 3-month period (February-March-April) in 2018, 2019, and 2020, respectively (the Supplementary Figure S1 reported the flow-chart of patient inclusion process). Baseline characteristics of the cohorts are shown in Table 1. The mean age of the subjects at the discharge was about 66 years (with 9–10% of individuals aged ≥85 years), and about 29% of them were women, without significant differences across years (chi square test for sex, *p*-value 0,65; ANOVA test for age, *p*-value 0.09). Regarding the type of AMI, during the 3 years of observation, the proportion of patients who experienced a ST-elevation myocardial infarction (STEMI) was 43–46%, while a Non-ST-elevation myocardial infarction (NSTEMI) occurred in 37–38% of patients. There was no clinically relevant difference across years also regarding the proportion of patients with at least one prescription of antiplatelet, antihypertensive, lipid-lowering agents, or antidiabetic drugs, or with a hospitalization for HF before the enrolment.

**TABLE 1:** Characteristics of the cohorts in the quarters February-March-April.

	2018	2019	2020
Number of subjects	2259	2383	1932
Age in years, mean (SD)	65.17 (14.05)	65.60 (13.78)	66.10 (13.25)
Age classes, %			
18–49 years	12.39	11.75	10.09
50–64 years	36.92	37.35	37.89
65–84 years	41.52	41.54	42.08
≥85 years	9.16	9.36	9.94
Female sex, %	28.82	29.63	28.36
Use of antiplatelet drugs, %	41.79	38.98	39.75
Use of antihypertensive drugs, %	51.44	52.62	53.47
Use of lipid-lowering drugs, %	41.92	42.13	42.91
Use of antidiabetic drugs, %	9.21	11.92	11.39
Hospitalization for heart failure, %	8.32	8.43	9.01
Type of AMI, %			
STEMI	43.96	42.97	46.27
NSTEMI	38.73	38.02	36.8
Treatment strategy of AMI patients, %			
Non-drug-eluting coronary artery stent(s)	0.27	0.17	0.21
CABG	15.71	17.42	15.89
PTCA	0.00	0.04	0.05

*Covariates have been collected at the index date or in a variable period of time before the index date; details are reported in the methods section.

AMI: acute myocardial infarction; SD: standard deviation; STEMI: ST-elevation myocardial infarction; NSTEMI: Non-ST-elevation myocardial infarction; CABG: coronary artery bypass graft surgery; PTCA: Percutaneous transluminal coronary angioplasty.

**FIGURE 1:**
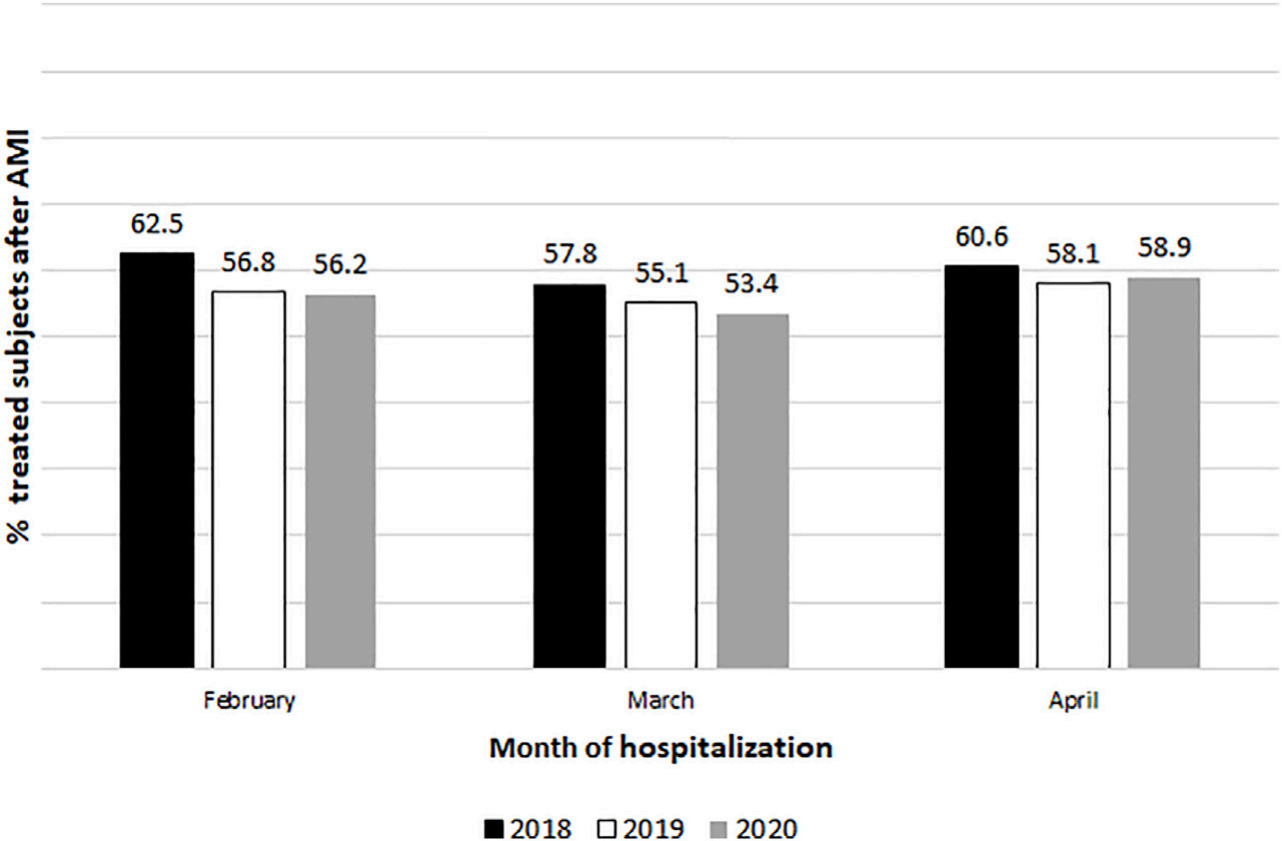
Percentage of subjects with a first prescription of beta-blockers in the month after the discharge for AMI hospitalization.

Figure 1 shows the percentage of subjects who started treatment with beta-blockers after AMI during each month of the quarter February-March-April 2020, compared with rates in the same months in the years 2019 and 2018. Overall in 2020, more than half of the individuals who were hospitalised for AMI received a prescription of beta-blockers within 1 month after the discharge. Rates were higher in 2018, then highlighting a decreasing trend over time. Among those who started the beta-blockers therapy, on average the treatment is started after 5 days from the discharge, with no difference among different periods (*p*-value 0.55) (Table 2). The sensitivity analysis when the follow-up period after the discharge was extended to 6 months, showed the same results with slightly higher rates for therapy initiation (Supplementary Figure S2). Stratified analyses are reported in the Supplementary Materials. Briefly, women reported lower rate of starting beta-blocker treatment compared with men, with more marked decreasing trends than those of the male cohort (Supplementary Figure S3). Regarding age (Supplementary Figure S4), the overall trend was maintained, with decreasing rates in 2020 compared to 2018, for subjects aged 50–84 years; in contrast, different trends were identified for the two extreme age groups: we observed lower percentages for subjects aged ≥85 years with respect to younger individuals (for instance, in April 2020, only 44% of individuals with AMI started a beta-blockers therapy within 1 month from the discharge compared with approximately 57% of individuals in February of the same year). On the other hand, younger patients showed higher treatment rates in 2020 than in previous years (in April 2020, about 64% of individuals with AMI started a beta-blockers treatment).

**TABLE 2:** Mean (SD) number of days between discharge and the start of treatment.

	February	March	April
2018	4.8 (8.1)	5.2 (8.2)	5.3 (8.5)
2019	5.7 (8.6)	4.5 (7.2)	5.5 (8.5)
2020	5.4 (8.0)	5.4 (8.2)	5.2 (8.2)

**FIGURE 2:**
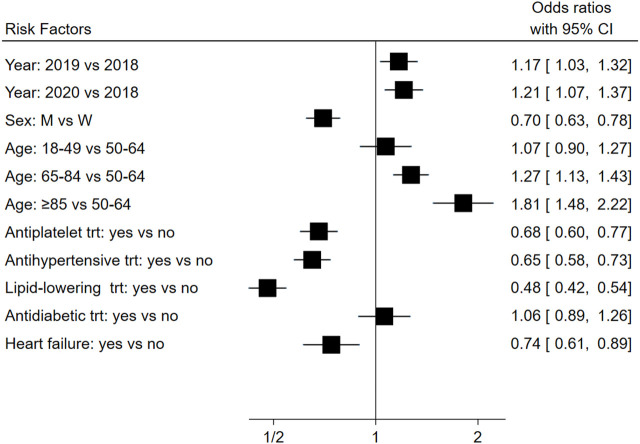
Association between several individuals’ covariates and the risk of not receiving a post-infarction beta-blocker therapy. *trt, treatment; CI, confidence interval.

Figure 2 reports the adjusted OR values and 95% confidence intervals for the probability of not receiving a post-infarction beta-blocker therapy. Overall, individuals hospitalised with AMI in the period February-March-April 2019 were 17% less likely to start the treatment (OR 1.17, 95% CI 1.03–1.32) compared with subjects hospitalised in the same period of the previous year. The probability of not being treated with beta-blockers was higher also in 2020 (OR 1.21, 95%CI 1.07–1.37) still compared with subjects hospitalised in 2018, despite the two estimates (in 2019 and in 2020) were not statistically different (*p*-value for comparison 0.71). Compared to women, men were 30% more likely to start the treatment (OR 0.70, 95% CI 0.63–0.78) after AMI. Increasing age was associated with significant increasing probability of not receiving a post-infarction beta-blocker therapy, with a risk that was almost double for very elderly subjects compared to individuals aged between 50 and 64 years at the time of the discharge (OR 1.27, 95% CI 1.13–1.43 and OR 1.81, 95% CI 1.48–2.22 for subjects aged 65–84 and ≥85 years, respectively). Having received other antiplatelet, antihypertensive, or lipid-lowering treatment in the 6 months prior to the hospitalization, or having been hospitalized for HF in the year prior to the hospital admission for AMI reduced the likelihood of not being treated with beta-blockers (OR 0.68, 95%CI 0.60–0.77; OR 0.65, 95%CI 0.58–0.73; OR 0.48, 95%CI 0.42–0.54; OR 0.74, 95%CI 0.61–0.89; respectively).

## Discussion

Beta-blockers represent one of the oldest classes of cardiovascular agents and have been considered as a cornerstone therapy for secondary cardiovascular prevention for the last 5 decades. Although their role has been downgraded in some patient subgroups, their use remains an important approach in cardiovascular prevention.

Our analysis of hospitalised subjects in the Italian Lombardy region shows that, in 2018, about 30% of patients did not receive this therapy after discharge for AMI, and that this proportion increased over time to almost half of the post-infarct subjects.

This trend is well evident in the 3 years considered. In particular, the last year evaluated, 2020, affected by the first wave of the COVID-19 pandemic, seems to be in line with previous years, without relevant changes in the percentages observed. In Italy, the national government declared the state of emergency on 31 January 2020, introduced measures for social distancing on 23 February 2020 and enforced a complete country lockdown on 9 March 2020. Hospitals and Emergency Departments were forced to rapidly adjust to this situation (Gatto et al., 2020; Indolfi and Spaccarotella, 2020). The complexity of managing an emergency situation may have influenced the approach to non-COVID patients. The unprecedented impact of COVID-19 pandemic across health care has been described in literature. Some studies reported that prescribing and dispensing of a wide variety of medications was affected during the pandemic, including health-critical medications (Frazer and Frazer, 2021). Healthcare professionals reported higher anxiety during the COVID-19 pandemic (Temsah et al., 2021), and this could have had an impact on their attitudes. At the outbreak of the pandemic, information about the virus, the syndrome it caused, and the effect of therapies was essentially non-existing. The literature reports examples of drugs that were empirically overused or underused during the first pandemic wave, because of the lack of conclusive evidence, in an attempt, for example, to minimise the risk of drug-drug interactions or the drug load in the elderly (Enners et al., 2021). Moreover, during the first wave, general practitioners suffered for an increased workload, and their daily activities were heavily occupied with caring for COVID patients. It is also important to consider that a considerable part of the physicians was in quarantine/self-isolation or tested positive for SARS-CoV-2 (Mahlknecht et al., 2022).

Our results seem to suggest that the prescription of beta-blockers after AMI has been slightly influenced by the pandemic, while indicating a trend of growing underuse of beta-blockers in secondary cardiovascular prevention. (Ellerbeck et al., 1995; Burwen et al., 2003). This was also confirmed by the logistic model that was implemented in this study; although it showed an increase in the probability of not starting the treatment after the discharge in 2020 compared to 2019, the estimate was not statistically different from the previous year, suggesting a tendency over time rather than an increase in risk attributable to COVID-19 pandemic. Our data, however, also suggest that the subgroups in which these drugs are least prescribed, i.e. women and the elderly, are those for whom the pandemic period has led to a more pronounced reduction in prescription rates.

A low beta-blocker prescription rate in women and the elderly has been already reported by literature. A descriptive cross-sectional study of women aged 65 and older who were 6–12 months post-AMI (Crane et al., 2006) reported that 26% were not treated with beta-blockers. In a community-based observational cohort study on 865 patients discharged from hospitals after first AMI (Wei et al., 2004), authors reported percentages of beta-blocker users decreasing from 62.5% among 50–59-year-old patients to less than 35% among over70 patients, and that women were less frequently treated compared to men. In a study on Canadian people aged 66 years or more who survived an acute MI, 48.6% were not dispensed a beta-blocker, with women and patients 85 years of age or more at greater risk of not receiving therapy (Rochon et al., 1999). In an evaluation of patients with AMI in Singapore, patients older than 65 years were less likely to receive beta-blockers compare to under65 patients (21.8% vs. 60.7%; *p* < 0.001) (Woon and Lim, 2003). Beta-blockers are listed among drugs often associated with under-prescription in the elderly (Marquez et al., 2017). This may reflect doctors’ attempt not to add another drug to patients who are already poly-treated, as elderly patients often are, and the exacerbation of this attitude during the pandemic may explain the sharp reduction in prescribing rate the over-80s, as well as the higher rate of treatment in younger individuals, where concomitant therapies are less likely. Regarding women, several studies have pointed out a sex-related gap in the treatment for AMI, that encompass timeliness in medical care, pharmacological treatments, and surgical procedures (Liakos and Parikh, 2018; Hao et al., 2019; Kuehnemund et al., 2021).

Other patient conditions may influence the decision to start beta-blocker treatment. In our study, the use of drugs for hypertension and dyslipidaemia, or the use of antiplatelet treatment were associated with a higher likelihood of starting therapy, suggesting greater awareness of the need to implement effective cardiovascular prevention. In contrast, the presence of antidiabetic treatment showed no impact, as already described in another study (Wei et al., 2004). Beta-blockers are fundamental in pharmacological management of chronic heart failure, however concerns and disagreement regarding their role in the treatment of decompensated HF during hospitalization are common in clinical practice (Zafrir and Amir, 2012). In this context, our findings showing an association between hospitalization for HF and a higher probability of starting therapy further supports clinicians’ awareness of the need for therapy.

Although some recent evidence has suggested that the use of other therapies, such as ACE inhibitors and statins, and advances in surgical therapy could make the use of beta-blockers unnecessary (Bangalore et al., 2012; Dondo et al., 2017; Holt et al., 2021), their prescription remains recommended by major guidelines (Amsterdam et al., 2014; Roffi et al., 2016; Ibanez et al., 2017). Overall, our data, especially in the light of the trend and of other evidence in the literature, seem to suggest that a significant proportion of post-infarction patients did not receive the recommended approach and suggest that prescribing practice after infarction needs to be improved. To optimise these efforts, some studies have attempted to understand what the barriers to prescribing these agents might be. In a qualitative interview study in Germany (Freier et al., 2020), the deviations from guidelines were because of side effects or patient intolerance. Some health care professionals questioned the benefits of medical secondary prevention for the oldest patients, or claimed that guidelines rarely address complicating factors such as comorbidities. In some cases, inaccurate or insufficient knowledge of the guidelines has also been reported (Kavookjian and Mamidi, 2008). The improvement of the knowledge and practice patterns of physicians could be achieved through multifaceted approaches aimed at increasing physician knowledge of clinical trial results through continuing-education programs and broader dissemination of practice guidelines (Kennedy and Rosenson, 1995). However, it is important to point out that a proportion of patients who did not start the treatment after the AMI may be those who, although being prescribed by the physician, did not collect the prescribed drugs at the pharmacy, although the nature of the data used in this study does not allow a distinction to be made between the two possibilities. In this case, the role of the general practitioner and the therapeutic alliance that is established with the patients, who must be educated on the importance of the therapy and the need to follow the treatment correctly, is fundamental.

This study has several limitations. First, in this study based on administrative health databases, clinical patient variables indicating conditions in which beta-blockers are not recommended (as respiratory disorders that cannot be traced with drug therapy or hypotension), or other explicit reasons for not prescribing beta-blockers as set by physicians, are not included. Furthermore, in the administrative database, heart failure or severe respiratory diseases could have been underdiagnosed. Nevertheless, administrative databases themselves are an element of strength, as they collect all the reimbursed drugs dispensed to all citizens covered by the NHS. Moreover, administrative data collection, managed at a regional level, is nationally standardized, extremely accurate, and commonly used for drug utilization and pharmacoepidemiological research (Trifiro et al., 2019). Another limitation of our analysis is the impossibility of assessing whether COVID-19 positivity may have influenced the prescribing decision. Although this may be an interesting aspect to investigate, the time window we defined for the year 2020 (February-May) was too early in the pandemic outbreak to provide robust data. Future studies are needed to understand whether and how the development of SARS-CoV-2 infection impacted beta-blockers prescribing.

## Conclusions

Our study found that the initiation of beta-blocker treatment after myocardial infarction showed a decreasing trend over time that does not seem to have been further affected by the first wave of the COVID-19 pandemic. Under-prescribing affected women more than men, and the elderly more than younger adults. Although some evidence seems to suggest that physicians pay more attention to subjects at higher cardiovascular risk, the substantial proportion of post-AMI patients who did not received the treatment and the decreasing trend in recent years emphasises the need for continuous monitoring of real-life data.

## Data Availability

Data are available from the authors upon reasonable request and with permission of Regione Lombardia.
