# Supplementary material for: Beta-blockers in post-acute myocardial infarction patients: Drug prescription patterns from 2018 to Italy’s first wave of the COVID-19 pandemic
Source: Front Pharmacol. 2022 Dec 7;13:1040710. doi: 10.3389/fphar.2022.1040710 (PMC9768333; doi:10.3389/fphar.2022.1040710)

**Supplementary materials**

**Beta-blockers in post-acute myocardial infarction patients: drug prescription patterns from 2018 to Italy’s first wave of the COVID-19 pandemic**

**Elena Olmastroni^1*^, Federica Galimberti^2^, Alberico L. Catapano^1,2^, Elena Tragni^1^, Manuela Casula^1,2^**

^1^ Epidemiology and Preventive Pharmacology Service (SEFAP), Department of Pharmacological and Biomolecular Sciences, University of Milan, Milan, 20133, Italy

^2^ IRCCS MultiMedica, Sesto San Giovanni (MI), 20099, Italy

**Supplementary Figure 1.** Flow-chart of the study.


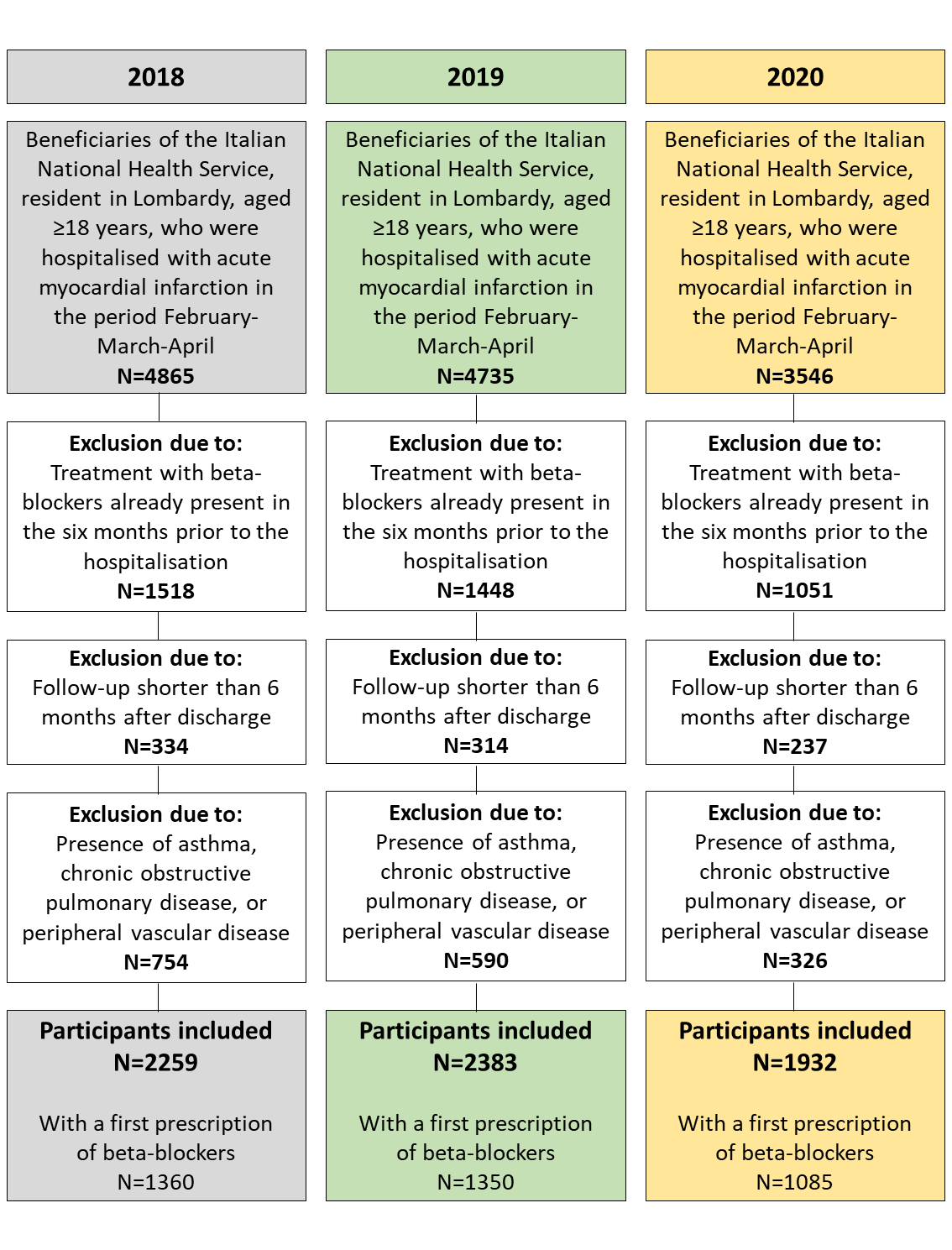


**Supplementary Figure 2.** Percentage of subjects with a first prescription of beta-blockers in the six months after the discharge for AMI hospitalization.


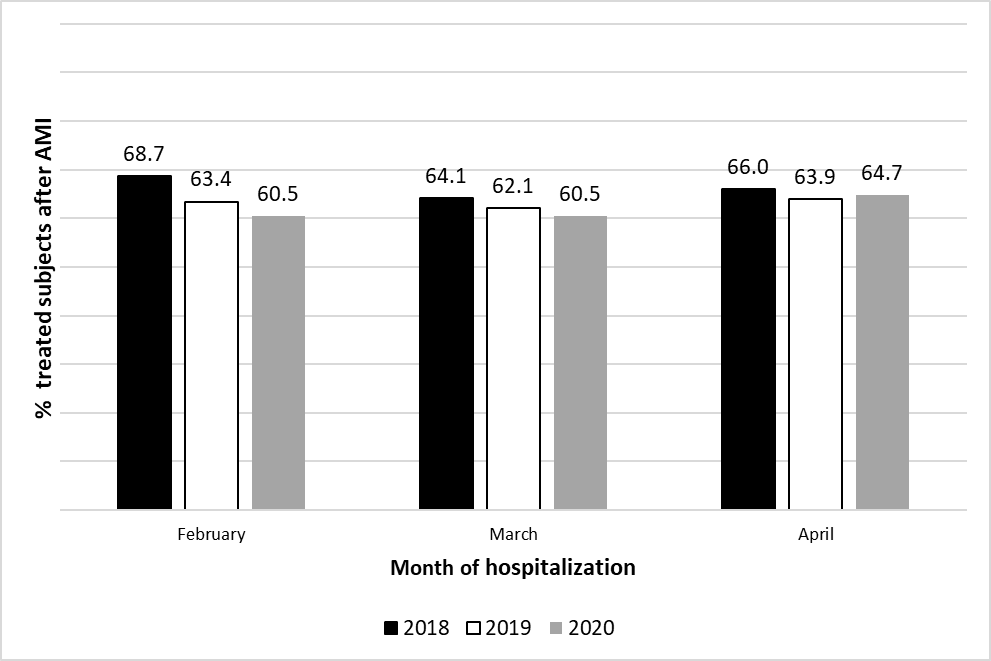


**Supplementary Figure 3.** Percentage of subjects with a first prescription of beta-blockers in the month after the discharge for AMI hospitalization by sex.

(A) Women


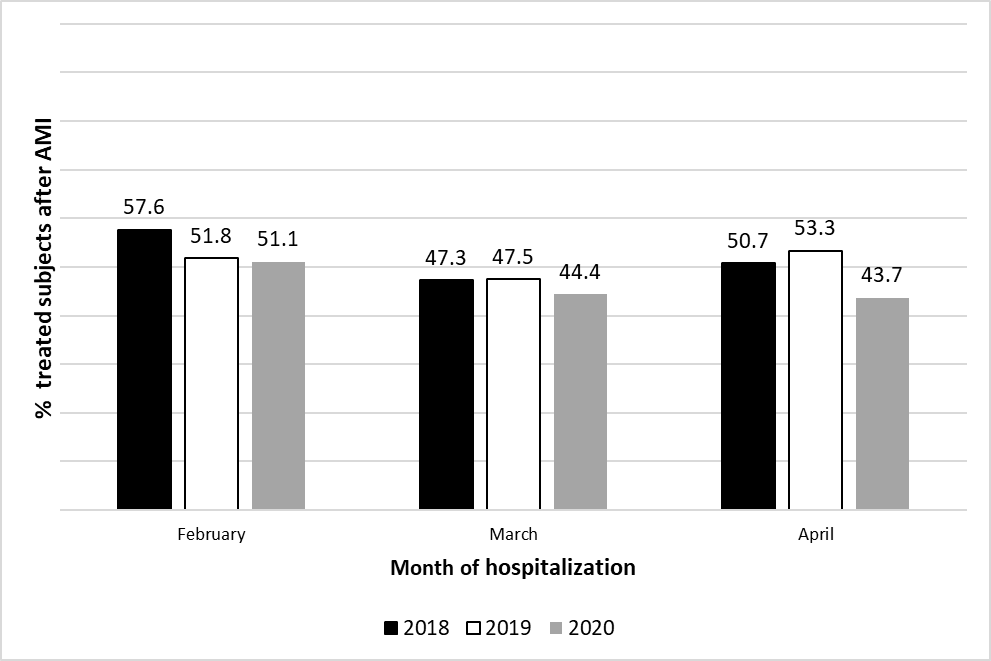


(B) Men


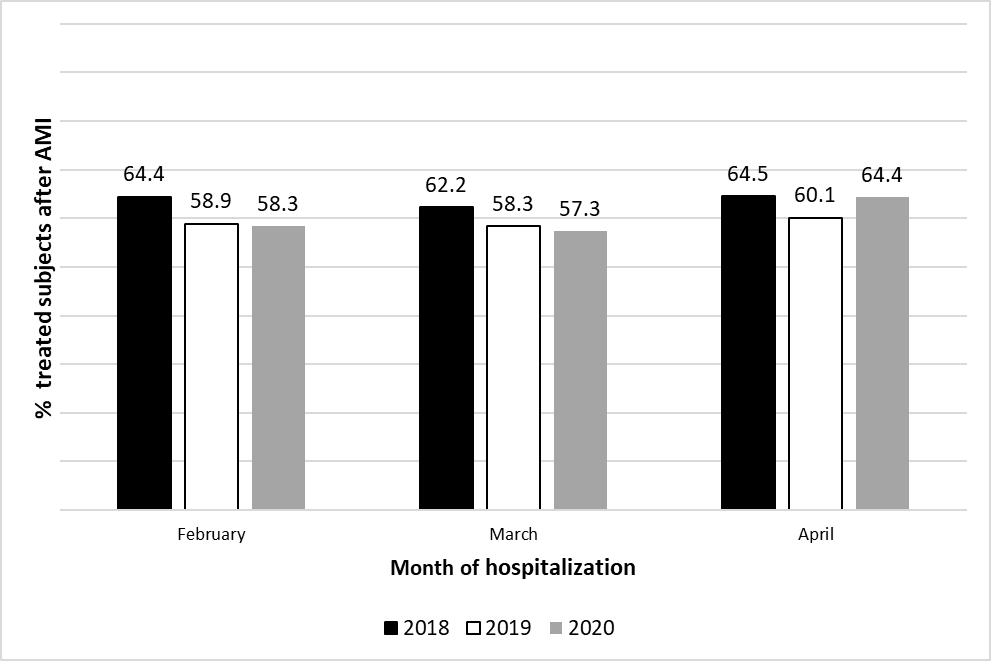


**Supplementary Figure 4.** Percentage of subjects with a first prescription of beta-blockers in the month after the discharge for AMI hospitalization by age classes.

(A) Individuals aged between 18-49 years


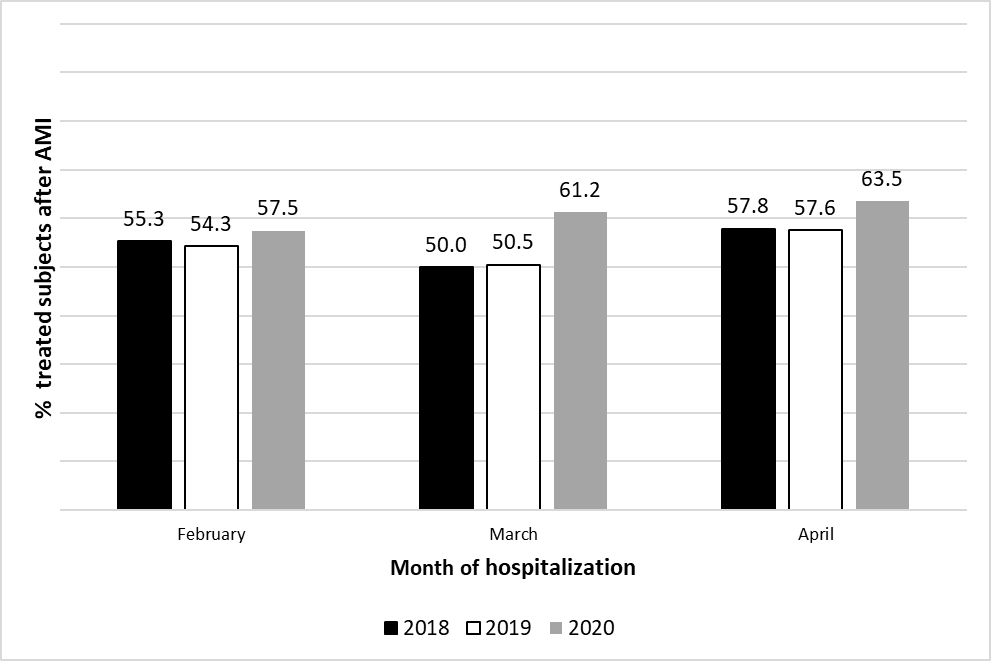


(B) Individuals aged between 50-64 years


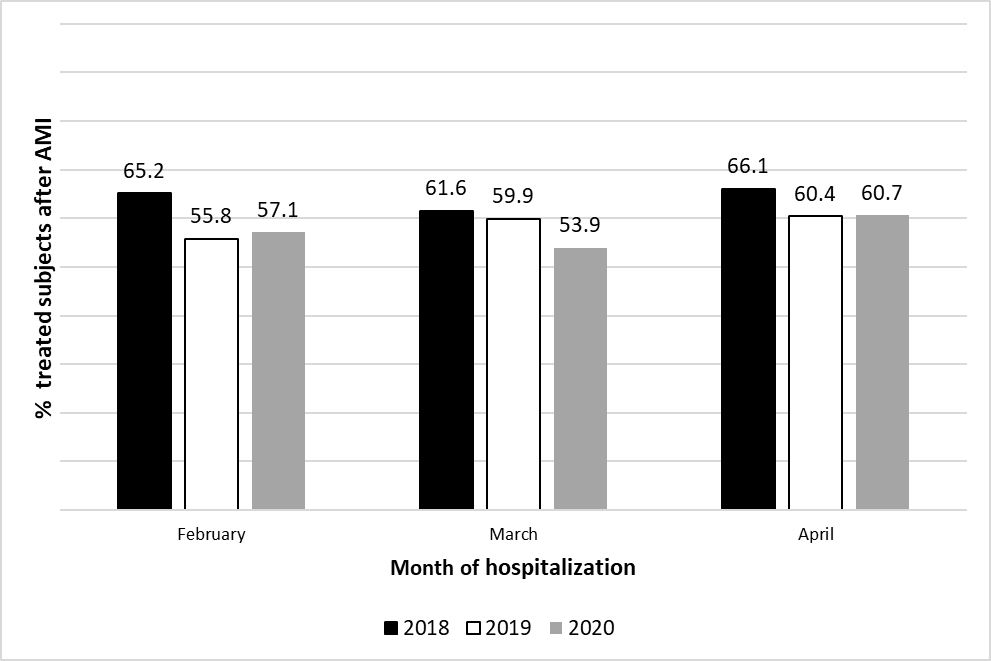


(C) Individuals aged between 65-84 years


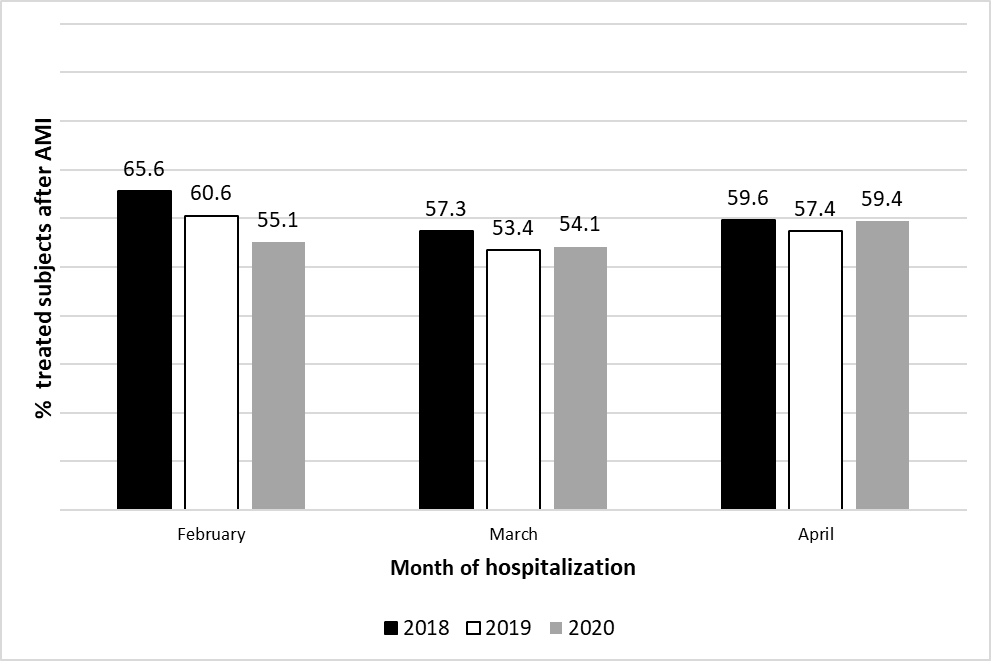


(D) Individuals aged ≥85 years


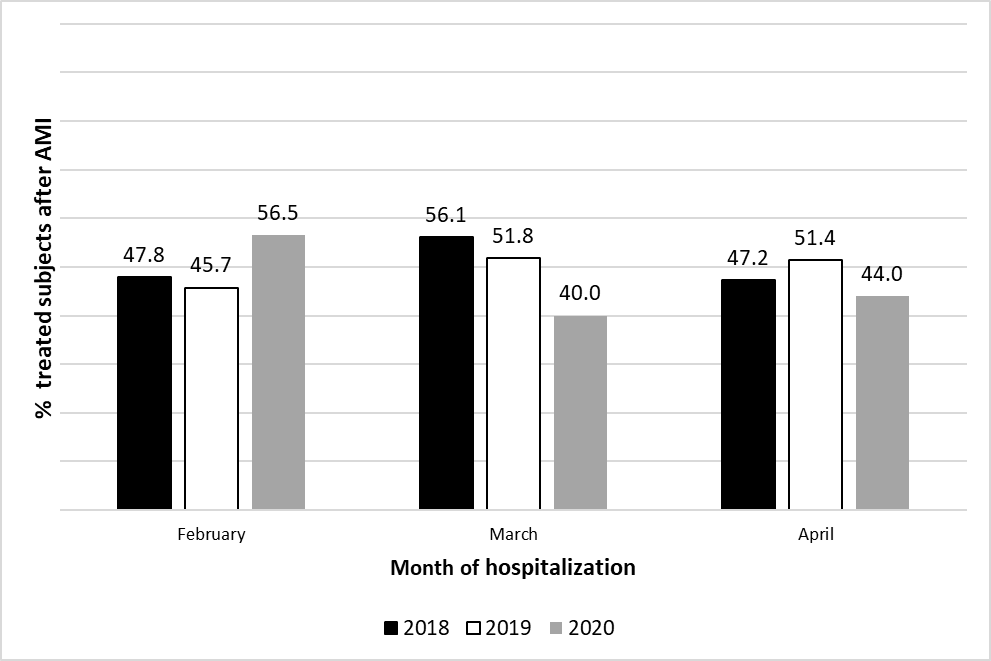

Supplement: Supplementary file 1 [file DataSheet1.docx]
